# Obesity and metabolic syndrome in patients with heart failure with preserved ejection fraction: a cross-sectional analysis of the Veradigm Cardiology Registry

**DOI:** 10.1186/s12902-024-01589-2

**Published:** 2024-05-01

**Authors:** Jay P Bae, Lee Kallenbach, David R Nelson, Kevin Lavelle, Jessamine P Winer-Jones, Machaon Bonafede, Masahiro Murakami

**Affiliations:** 1 Eli Lilly and Company, Indianapolis, USA; 2 Veradigm, Chicago, USA

**Keywords:** Heart failure preserved ejection fraction, Left ventricular ejection fraction, Obesity, Metabolic syndrome

## Abstract

**Background:**

The proportion of heart failure patients with preserved ejection fraction has been rising over the past decades and has coincided with increases in the prevalence of obesity and metabolic syndrome. The relationship between these interconnected comorbidities and heart failure with preserved ejection fraction (HFpEF) is still poorly understood. This study characterized obesity and metabolic syndrome among real-world patients with HFpEF.

**Methods:**

We identified adults with heart failure in the Veradigm Cardiology Registry, previously the PINNACLE Registry, with a left ventricular ejection fraction measurement ≥ 50% between 01/01/2016 and 12/31/2019. Patients were stratified by obesity diagnosis and presence of metabolic syndrome (≥ 3 of the following: diabetes, hypertension, hyperlipidemia, and obesity). We captured baseline demographic and clinical characteristics and used multivariable logistic regression to examine the odds of having cardiac (atrial fibrillation, coronary artery disease, coronary artery bypass surgery, myocardial infarction, and stroke/transient ischemic attack) and non-cardiac (chronic kidney disease, chronic liver disease, and peripheral artery disease) comorbidities of interest. The models adjusted for age and sex, and the main covariates of interest were obesity and metabolic burden score (0–3 based on the presence of diabetes, hypertension, and hyperlipidemia). The models were run with and without an obesity*metabolic burden score interaction term.

**Results:**

This study included 264,571 patients with HFpEF, of whom 55.7% had obesity, 52.5% had metabolic syndrome, 42.5% had both, and 34.3% had neither. After adjusting for age, sex, and burden of other metabolic syndrome-associated diagnoses, patients with HFpEF with obesity had lower odds of a diagnosis of other evaluated comorbidities relative to patients without obesity. The presence of metabolic syndrome in HFpEF appears to increase comorbidity burden as each additional metabolic syndrome-associated diagnosis was associated with higher odds of assessed comorbidities except atrial fibrillation.

**Conclusion:**

Obesity was common among patients with HFpEF and not always co-occurring with metabolic syndrome. Multivariable analysis suggested that patients with obesity may develop HFpEF in the absence of other driving factors such as cardiovascular disease or metabolic syndrome.

**Supplementary Information:**

The online version contains supplementary material available at 10.1186/s12902-024-01589-2.

## Background

Historically, heart failure (HF) has been associated with reduced ejection fraction (HFrEF); however, the incidence of heart failure with preserved ejection fraction (HFpEF) has been on the rise and may account for over half of newly diagnosed HF patients [1, 2]. In contrast to patients with HFrEF, HFpEF occurs more commonly in women and the elderly and is less responsive to most currently available medical therapy options, though some newer treatments show promise in this population [3–6].

Metabolic syndrome is a cluster of clinical measures, including increased waist circumference, elevated triglycerides, reduced high-density lipoprotein cholesterol, elevated blood pressure, and elevated fasting glucose, which are associated with an increased risk of cardiovascular disease [7]. Insulin resistance was initially thought to be the driver of metabolic syndrome [8]; however, newer research has suggested that metabolic syndrome derives from a complex interplay between obesity, hypertension, hyperlipidemia, and insulin resistance [9]. While abdominal obesity is one of the criteria for metabolic syndrome [7], some individuals exhibit metabolically healthy obesity in that they meet the body mass index (BMI) criteria for obesity in the absence of other metabolic comorbidities [10]. Despite both obesity and metabolic syndrome being risk factors for HFpEF [11–13], some evidence suggests that metabolically healthy obesity is not associated with an increased risk of HF [14, 15].

While obesity is prevalent among patients with HFpEF, there is conflicting evidence on whether it is a driver or bystander of HFpEF development and progression. Furthermore, we do not fully understand the clinical characteristics of these subpopulations. This cross-sectional analysis of the Veradigm Cardiology Registry sought to characterize profiles of patients with HFpEF stratified by obesity status, defined by BMI, and by metabolic syndrome status, defined by diagnosis codes, and characterize differences in patient profiles. We explored the interaction between obesity, metabolic syndrome, and the presence of other comorbidities in patients with HFpEF.

## Methods

### Data source

This retrospective analysis leveraged the Veradigm Cardiology Registry, previously the PINNACLE Registry. This registry was established in 2008 by the American College of Cardiology’s National Cardiovascular Data Registry to collect data on U.S. outpatient cardiovascular care of patients with HF, coronary artery disease, atrial fibrillation, or hypertension [16] and has been used in several studies of HF [17, 18]. Contributing clinical practices contribute longitudinal patient data, leveraging a technology platform to extract and standardize clinical data from electronic health records. Extracted data includes detailed information on symptoms, signs, medication prescribing, procedures, and outcomes [19, 20].

The dataset available for research contains only de-identified data as per the de-identification standard defined in Section § 164.514(a) of the Health Insurance Portability and Accountability Act of 1996 (HIPAA) Privacy Rule. As a noninterventional, retrospective database study using data from a certified HIPAA–compliant de-identified research database, approval by an institutional review board was not required.

### Study cohort

We identified adults, age 18 and older, in the Veradigm Cardiology Registry with at least one diagnosis of HF, at least one visit with a cardiologist, and a left ventricular ejection fraction (LVEF) measurement of ≥ 50% documented by a cardiologist between January 1, 2016, and December 31, 2019. The index date was the date of the first qualifying LVEF measurement. Patients were required to have a BMI within 365 days of the index date, non-missing gender on the index date, and no evidence of an LVEF measurement ≤ 40% within 365 days of the index date.

For the obesity analysis, patients were stratified by the presence or absence of a BMI ≥ 30, determined by the BMI value observed on the date closest to the index date, with ties going to dates occurring prior to the index date. Patients with obesity were further segmented by obesity class: class 1 (30 ≤ BMI < 35), class 2 (35 ≤ BMI < 40), and class 3 (BMI ≥ 40).

For the metabolic syndrome analysis, patients were coded as having metabolic syndrome if they had a prior diagnosis of at least three of the following four conditions: diabetes, hypertension, hyperlipidemia, and obesity.

### Study variables

We captured age, sex (male or female), and race/ethnicity (non-Hispanic White, non-Hispanic Black, Hispanic, or other/unknown) on the index date. To comply with de-identification standards, patient age was truncated at 80 years old in the registry. In addition to the comorbidities used to define the study cohorts, we also captured prior diagnosis of atrial fibrillation, coronary artery disease, coronary artery bypass surgery, chronic kidney disease, chronic liver disease, myocardial infarction, peripheral artery disease, and stroke/transient ischemic attack (TIA) using the data fields defined in the Registry [20].

Where available, we also captured select laboratory results and vitals, including systolic blood pressure, diastolic blood pressure, total cholesterol, high-density lipoprotein (HDL) cholesterol, low-density lipoprotein (LDL) cholesterol, triglycerides, estimated glomerular filtration rate (eGFR), and New York Heart Association Functional Classification. For each measure, we took the value observed on the date closest to the index date, with ties going to dates occurring prior to the index date.

### Data analysis

Categorical variables were reported as counts and percentages, while continuous variables were reported as medians and interquartile ranges (IQR).

We used multivariable logistic regression to help understand the relationship between obesity, metabolic syndrome, and the presence of other comorbidities in patients with HFpEF. We evaluated 8 independent generalized linear models, each using a logit link and binomial distribution to estimate the adjusted odds of patients having each comorbidity of interest. The comorbidities of interest were atrial fibrillation, coronary artery disease, coronary artery bypass surgery, chronic kidney disease, chronic liver disease, myocardial infarction, peripheral artery disease, and stroke/TIA.

Comorbid obesity was the independent variable of interest in all models. Models also adjusted for age (18–64, 65–79, and 80+), sex (male and female), and metabolic burden score (0–3). We defined the metabolic burden score as an ordinal variable with a value of 0, 1, 2, or 3 depending on the number of additional metabolic syndrome-associated diagnoses (diabetes, hypertension, and hyperlipidemia) identified in the patient record. Missing values were not imputed. To account for a possible interaction between the obesity and metabolic burden score, we ran all 8 models with and without an interaction term. Results of the logistic regression are reported as odds ratios with an associated 95% confidence interval (CI).

Data processing was conducted with SQL: ANSI (Dec 2021 release) within the Snowflake Data platform using Dbeaver Community version 22.2.0. The logistic regression was conducted in R version 4.2.2.

## Results

**Fig. 1:**
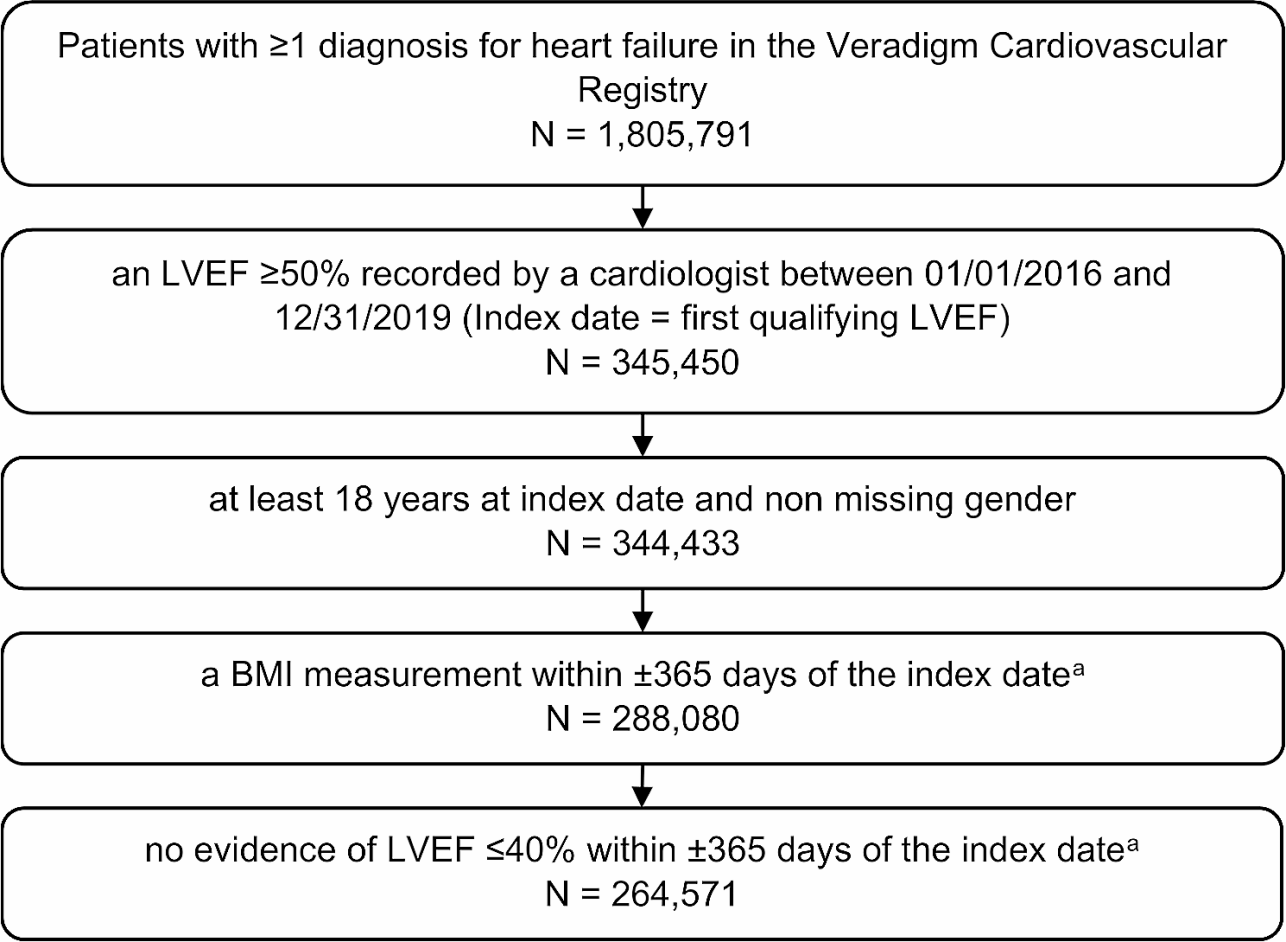
Patient Selection. ^a^This included the 365 days preceeding the index date and the 365 days following the index date but did not require continuous follow-up during this window. BMI, body mass index; LVEF, left ventricular ejection fraction

**Fig. 2:**
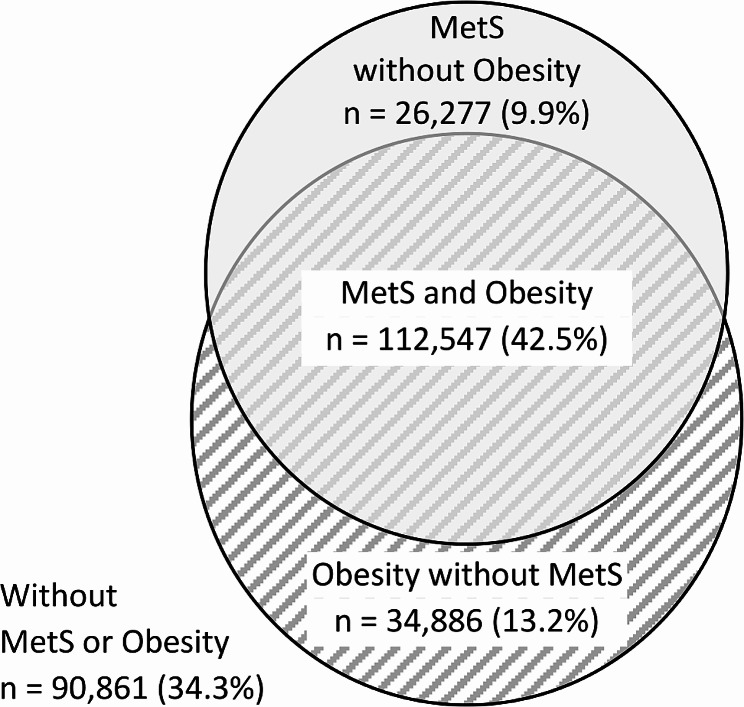
Overlap of Obesity and Metabolic Syndrome Among Patients with Heart Failure with Preserved Ejection Fraction^a^. ^a^Obesity-related heart failure with preserved ejection fraction is indicated by the diagonal shading. MetS, metabolic syndrome

We identified 264,571 patients with HFpEF in the Veradigm Cardiology Registry that met the patient selection criteria (Fig. 1). The median (IQR) age of patients with HFpEF was 76 (67–80) years (Table 1). Overall, 53.1% were female, 59.6% were non-Hispanic White, and 7.8% were non-Hispanic Black. Among all patients with HFpEF, 55.7% had a BMI ≥ 30 (mean [SD]: 32.8 [8.3]), indicating obesity-related HFpEF, while 89.4% had a diagnosis of hypertension, 71.4% had a diagnosis of dyslipidemia, and 36.8% had a diagnosis of diabetes. Notably, 52.5% of the overall HFpEF population had metabolic syndrome, 42.5% had both obesity and metabolic syndrome, and 34.3% had neither obesity nor metabolic syndrome (Fig. 2).

**Table 1:** Patient Characteristics

	All HFpEF Patients		Obesity Status			
			HFpEF with Obesity		HFpEF without Obesity	
	*N* = 264,571		*N* = 147,433		*N* = 117,138	
Age (median, IQR)	76	(67–80)	72	(64–80)	80	(72–80)
Age Group (N, %)						
18–64	53,720	20.3%	39,469	26.8%	14,251	12.2%
65–79	86,332	32.6%	56,884	38.6%	29,448	25.1%
80+	124,519	47.1%	51,080	34.6%	73,439	62.7%
Sex, Female (N, %)	140,499	53.1%	85,148	57.8%	55,351	47.3%
Race/Ethnicity (N, %)						
Non-Hispanic White	157,768	59.6%	85,027	57.7%	72,741	62.1%
Non-Hispanic Black	20,481	7.7%	14,375	9.8%	6,106	5.2%
Hispanic	11,652	4.4%	6405	4.3%	5,247	4.5%
Other/Unknown	74,670	28.2%	41,626	28.2%	33,044	28.2%
BMI (mean, SD)	32.8	8.3	38.2	7.0	26.0	3.4
Metabolic Syndrome Components (N, %)						
Diabetes	97,409	36.8%	64,635	43.8%	32,774	28.0%
Dyslipidemia	189,002	71.4%	105,347	71.5%	83,655	71.4%
Hypertension	236,493	89.4%	134,048	90.9%	102,445	87.5%
Obesity	147,433	55.7%	147,433	100%	-	0.0%

BMI, body mass index; HFpEF, heart failure with preserved ejection fraction; IQR, interquartile range; SD, standard deviation.

Patients with obesity-related HFpEF were younger (median age: 72 years vs. 80 years), female (57.8% vs. 47.3%), and a higher percentage were non-Hispanic Black (9.8% vs. 5.2%) than patients with HFpEF without obesity (Table 1). Within this subgroup of patients with obesity-related HFpEF, the same relationships were maintained with obesity class. Higher obesity class correlated with younger age, a higher percentage of female individuals, and a higher percentage of non-Hispanic Black individuals (Table 2). In particular, the percentage of patients at least 80 years old decreased from 45.9% among patients with class 1 obesity to 19.6% among patients with class 3 obesity, and the median age decreased from 75 years to 68 years.

**Table 2:** Patient characteristics by obesity class or metabolic syndrome status

	Metabolic Syndrome Status				Obesity Class					
	HFpEF with Metabolic Syndrome		HFpEF without Metabolic Syndrome		HFpEF with Class 1 Obesity		HFpEF with Class 2 Obesity		HFpEF with Class 3 Obesity	
	*N* = 138,824		*N* = 125,747		*N* = 147,433		*N* = 117,138		*N* = 138,824	
Age (median, IQR)	74	(66–80)	80	(68–80)	75	(67–80)	72	(65–80)	68	(60–75)
Age Group (N,%)										
18–64	29,324	21.1%	24,396	19.4%	11,690	19.1%	9633	24.5%	18,146	38.7%
65–79	53,081	38.2%	33,251	26.4%	21,400	35.0%	15,909	40.4%	19,575	41.7%
80+	56,419	40.6%	68,100	54.2%	28,092	45.9%	13,793	35.1%	9195	19.6%
Sex, Female (N,%)	74,053	53.3%	66,446	52.8%	29,741	48.6%	22,287	56.7%	33,120	70.6%
Race/Ethnicity (N,%)										
Non-Hispanic White	80,873	58.3%	76,895	61.2%	36,739	60.0%	23,034	58.6%	25,254	53.8%
Non-Hispanic Black	12,653	9.1%	7828	6.2%	4327	7.1%	3624	9.2%	6424	13.7%
Hispanic	6754	4.9%	4898	3.9%	2869	4.7%	1732	4.4%	1804	3.8%
Other/Unknown	38,544	27.8%	36,126	28.7%	17,247	28.2%	10,945	27.8%	13,434	28.6%
BMI (mean, SD)	36.0	7.8	29.2	7.5	32.3	1.4	37.3	1.4	46.5	5.3
Metabolic Syndrome Components (N,%)										
Diabetes	89,628	64.6%	7781	6.2%	23,903	39.1%	17,648	44.9%	23,084	49.2%
Dyslipidemia	128,257	92.4%	60,745	48.3%	45,585	74.5%	28,553	72.6%	31,209	66.5%
Hypertension	137,087	98.7%	99,406	79.1%	55,281	90.4%	36,003	91.5%	42,764	91.2%
Obesity	112,547	81.1%	34,886	27.7%	61,182	100%	39,335	100%	46,916	100%

BMI, body mass index; HFpEF, heart failure with preserved ejection fraction; IQR, interquartile range; SD, standard deviation.

Among patients with obesity-related HFpEF, 90.9% had a diagnosis of hypertension, 71.5% had a diagnosis of dyslipidemia, and 43.8% had a diagnosis of diabetes (Table 1). When examined by obesity class, the prevalence of hypertension was similar across classes, ranging from 90.4 to 91.5% (Table 2). The prevalence of dyslipidemia decreased from 74.5% in class 1 obesity to 66.5% in class 3 obesity, whereas the prevalence of diabetes increased from 39.1% in class 1 obesity to 49.2% in class 3 obesity.

Among patients with HFpEF and metabolic syndrome, the most common contributing factors were hypertension (98.7%) and dyslipidemia (92.4%), while 64.6% had a diagnosis of diabetes and 81.1% had a BMI indicating obesity (Table 2). By comparison, among patients with HFpEF without metabolic syndrome, diabetes and obesity were present in only 6.2% and 27.7% of patient records, respectively.

**Fig. 3:**
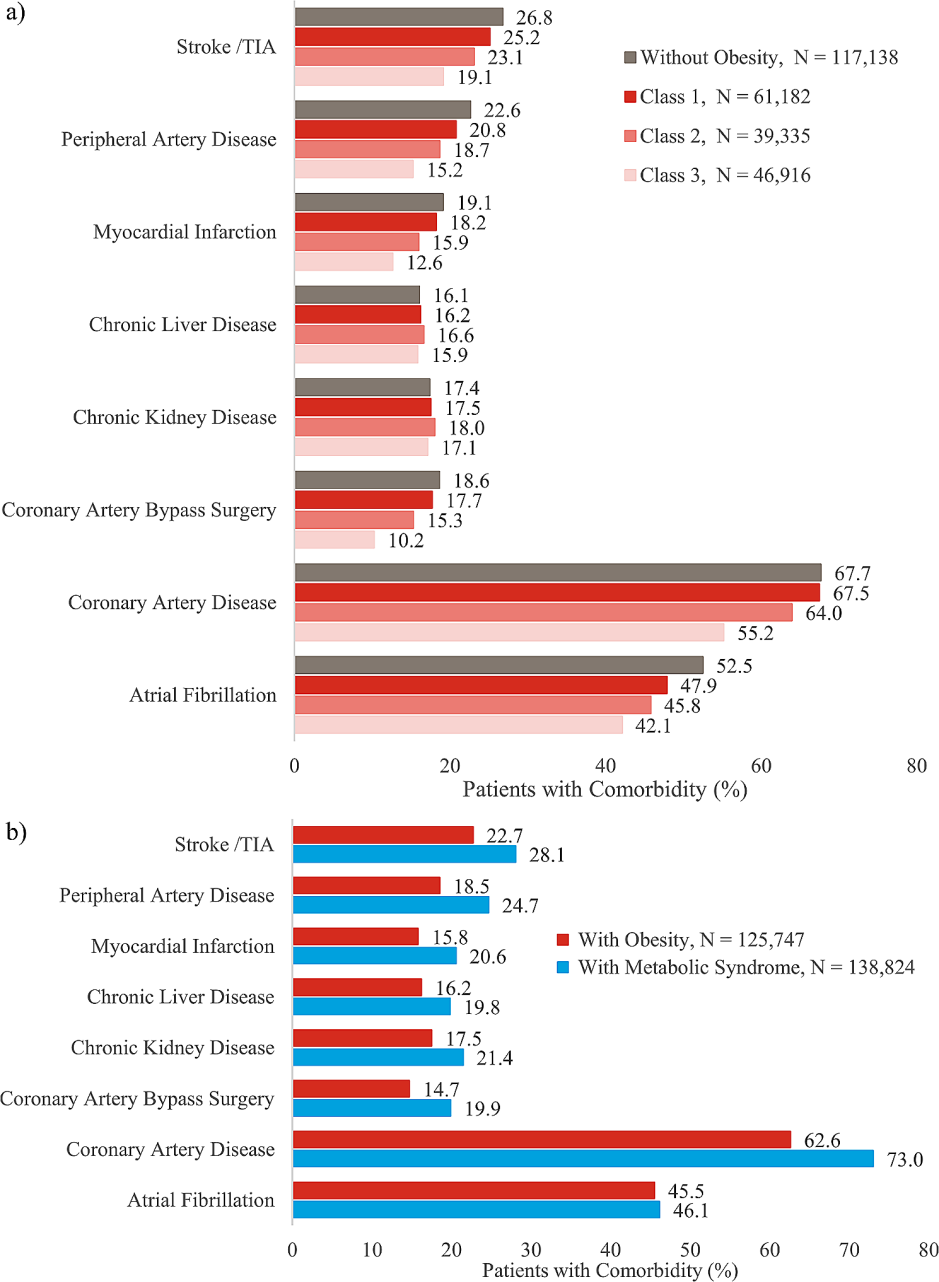
Comorbidities Among HFpEF Patients (a) stratified by Obesity Class or (b) Obesity or Metabolic Syndrome. HFpEF, heart failure with preserved ejection fraction; TIA, transient ischemic

In general, there appeared to be a negative correlation between obesity class and the prevalence of other cardiac conditions, such as coronary artery disease, atrial fibrillation, and stroke/TIA (Fig. 3a and Additional File 1). However, the prevalence of chronic kidney disease and chronic liver disease appeared to be independent of obesity class. Notably, with the exception of atrial fibrillation, the prevalence of both cardiac and non-cardiac conditions was higher in patients with HFpEF and metabolic syndrome compared to those with obesity-related HFpEF (Fig. 3b and Additional File 1).

Select vitals and laboratory results are reported in Additional File 1. Mean systolic and diastolic blood pressure ranged between 127.6–130.8 and 70.6–74.8, respectively, across the cohorts and were highest among HFpEF patients with class 3 obesity and lowest among HFpEF patients without obesity. Among the roughly one-third of patients with available labs, mean total cholesterol ranged from 153.2 to 159.2, mean HDL cholesterol ranged from 46.0 to 51.2, mean LDL cholesterol ranged from 79.9 to 85.7, and mean triglycerides ranged from 115.0 to 149.8. Among the 27.9% of patients with eGFR reported, all cohorts had a mean value of less than 60.

**Fig. 4:**
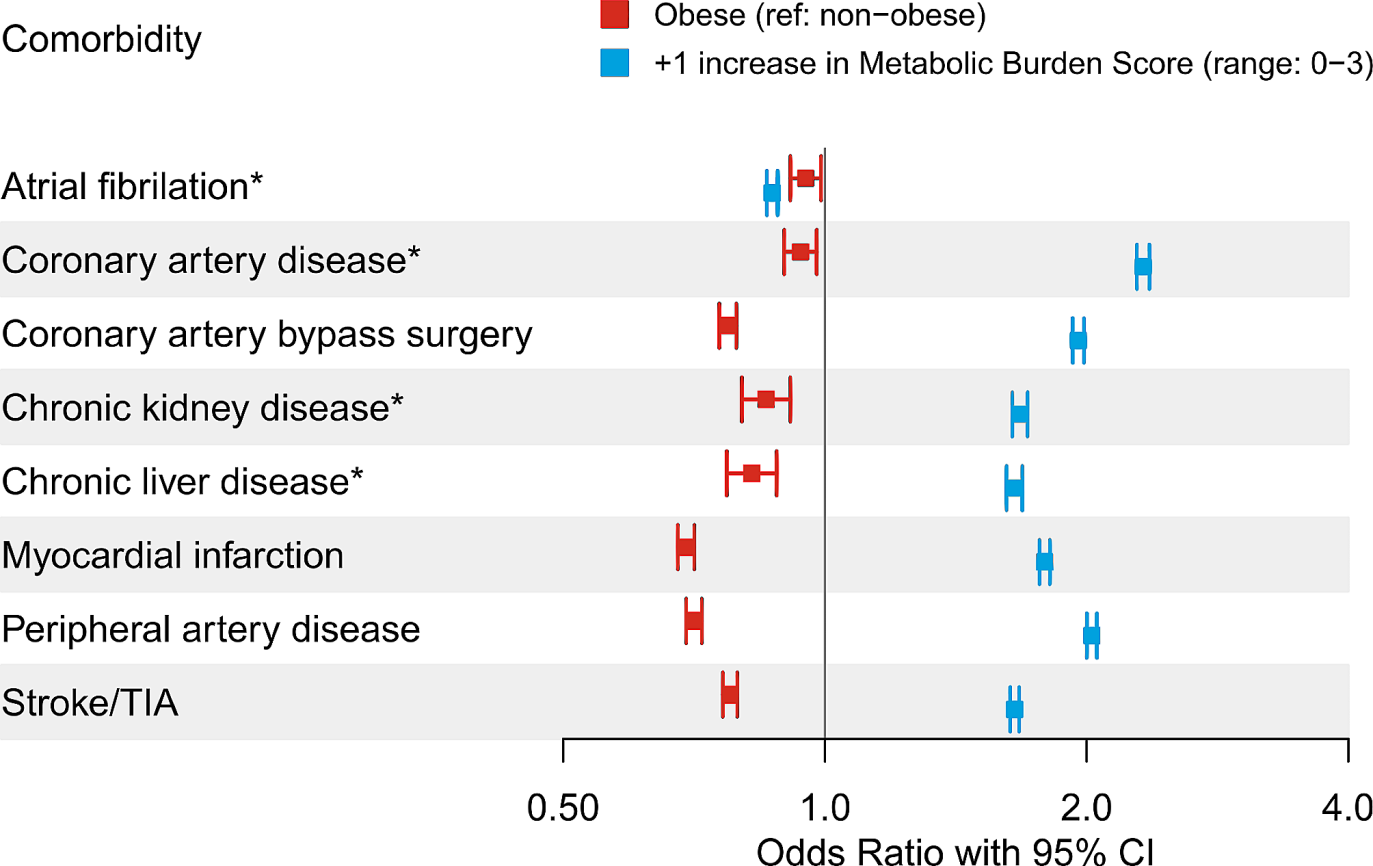
Odds of Select Comorbidities. *interaction term between obesity and metabolic burden score was significant. Reported odd ratio (OR) and confidence intervals (CI) for indicated comorbidities came

After adjusting for age, sex, and burden of other metabolic syndrome-associated diagnoses, patients with obesity-related HFpEF had lower odds of having a diagnosis of other evaluated comorbidities relative to patients with HFpEF and a BMI < 30 (Fig. 4). By contrast, each 1-point increase in metabolic burden score was associated with significantly higher odds of all comorbidities except atrial fibrillation. With two exceptions, older age, and male sex were associated with higher odds of comorbidities (Additional File 2).

## Discussion

In this analysis of the Veradigm Cardiology Registry, obesity and metabolic syndrome were common among patients with HFpEF. The subset of patients with obesity-related HFpEF tended to be younger, female, and non-Hispanic Black compared to those without comorbid obesity. Patients with obesity-related HFpEF had lower odds of comorbidities than patients with HFpEF without obesity. By contrast, a higher metabolic burden score was associated with higher odds of all comorbidities except atrial fibrillation. This suggests patients with obesity may develop HFpEF in the absence of other driving factors, such as cardiovascular disease or metabolic syndrome.

In this study, coronary artery disease (CAD) was the comorbidity most strongly associated with higher metabolic burden in HFpEF. This is consistent with previous smaller clinical studies which have shown a higher prevalence of diabetes and dyslipidemia (as indicated by statin use) in HFpEF patients with comorbid CAD [21, 22]. Concerningly, these studies also found that CAD is also associated with poorer outcomes among patients with HFpEF [21, 22]. HFpEF with CAD is considered to be a distinct phenotype from HFpEF with obesity [23–25], but our analysis supports an overlap with other aspects of metabolic syndrome.

The majority of patients with obesity-related HFpEF in this study also met the criteria for metabolic syndrome. This is consistent with other studies, which have found a high degree of overlap between HF, obesity, and metabolic syndrome [26–28]. Obesity is an established risk factor for HF overall and HFpEF in particular [29, 30]; however, it is unclear whether it should be considered a risk factor independent of metabolic syndrome. While comorbid metabolic syndrome is associated with a higher risk of hospitalization for HF among patients with HFpEF [26], obesity may be protective against the development of HF in the absence of other metabolic factors [14, 15]. The findings of this study support exploring obesity as an independent driver of HFpEF rather than an outcome of cardiovascular comorbidities.

A comparative clinical assessment of patients with obesity-related HFpEF and those with non-obese HFpEF found significant differences in cardiac structure, function, and hemodynamics between cohorts [27]. Separately, among patients with HFpEF who appeared metabolically healthy, indicators of diastolic dysfunction were observed more frequently among individuals with higher BMI [31]. These findings, along with our own, suggest that efforts to identify subtypes of HFpEF may benefit from further refining the obesity-related HFpEF population by the presence of comorbid metabolic syndrome.

A review of observational studies of bariatric surgery among patients with obesity-related HF found that bariatric surgery was associated with improved quality of life, reduced readmission rates for HF, and improved New York Heart Association functional class [32]. While the studies included in the review article were not specific to HFpEF, a subsequent study found that bariatric surgery-induced body mass reduction was associated with improved functional scores, improved diastolic function, decreased left ventricular mass, and reduced resting heart rate in 12 women with obesity-related HFpEF [33]. This potential connection between body mass reduction and improved clinical outcomes for patients with obesity-related HFpEF has been tested in randomized controlled trials of the glucagon-like peptide 1 agonist and supported by the results of a recent study demonstrating that treatment with semaglutide 2.4 mg weekly led to larger reductions in symptomatic and physical burdens, greater improvements in functional capacity than placebo in patients with HFpEF and obesity [34]. Moreover, one randomized controlled trial of the glucagon-like peptide 1 receptor/glucose-dependent insulinotropic polypeptide receptor agonist, tirzepatide, is currently underway [35–38]. Based on the outcomes of these trials, further refinement may be necessary to determine if outcomes among patients with obesity-related HFpEF are contingent on the presence of comorbid metabolic syndrome.

Another prevailing theory is that obesity-associated HFpEF is driven by a comorbidity-induced systemic proinflammatory state [39–41]. For example, the microRNA miR-181c is differentially expressed in patients with diabetes and comorbid HFpEF, and overexpression of miR-181c has been associated with proinflammatory conditions [42]. Similarly, testosterone has demonstrated anti-inflammatory effects [43], and testosterone deficiency has been associated with HFpEF in males with a cardio-metabolic profile [44]. This proinflammatory state contributes to impaired autophagy of vascular smooth muscle cells and endothelial cells, increased interstitial fibrosis, and stiff cardiomyocytes [39, 45]. It is currently unclear whether targeting these comorbidity-induced inflammatory pathways could result in improved outcomes for patients with HfpEF, but it is an area of active research [46].

Another factor complicating the interpretation of this study is the interaction between age and BMI. Heart failure tends to be a disease of older adults; however, in this study, median patient age was lower among patients with obesity-related HFpEF. With the data available, we were unable to determine if this was due to earlier onset of HFpEF among patients with obesity or higher mortality among older adults with obesity, leading to a selection bias towards younger patients with obesity-related HFpEF. There are also questions about what is the appropriate BMI cut-off for older adults. For example, a recent analysis found that the optimal BMI for women over 65 may be above the cut-off of 30, commonly used to define obesity [47]. While BMI is a widely available metric in structured clinical records, new research suggests it may not be the ideal measurement for assessing health risks associated with excess body weight [48]; however, until there is a fundamental shift in routine data collection, BMI remains the most widely used metric for assessing obesity in retrospective observational research.

### Limitations and strengths

This study is subject to several limitations inherent to the selected data source. First, the Veradigm Cardiology Registry was originally developed as a large outpatient quality improvement program based on voluntary participation and is, therefore, not a random sample of US cardiology practices. This may also impact the composition of patients included in the registry, as not all individuals with heart failure have access to specialty care. In addition, this data source should not be used to assess severe patient outcomes due to incomplete or lacking capture of key measures, such as hospitalizations and deaths.

Second, our study was not designed to identify an incident HFpEF population. Therefore, the documentation of a diagnosis should be interpreted as the patient had a diagnosis of the comorbidity of interest prior to the index date, not prior to the diagnosis of HFpEF. Laboratory results were only available for a subset of patients. Therefore, caution should be taken when assessing values overall and comparing values between cohorts, as there may be significant bias in which patients are being tested. For example, patients with suspected kidney disease may be more likely to have a documented eGFR value. As a result, laboratory results were not included as model covariates. Race and ethnicity were also not included as covariates due to the high degree of missingness.

Finally, we used BMI and diagnosis history as proxies for the standard clinical approach to assessing obesity and metabolic disease status [7]; however, these proxies are imprecise and may introduce bias into our analysis. Specifically, assessment for metabolic syndrome typically uses waist circumference or waist-to-hip ratio to identify at-risk individuals. The use of BMI as a proxy for obesity may overestimate the number of people with metabolically-relevant obesity and reduce the effect size of obesity as a predictor.

The strengths of this study include the size of the study population and the nature of the Veradigm Cardiology Registry. Specifically, patients in the registry are geographically distributed, sourced from small and large clinical practices, and come from a range of insurance plans, including Medicare, Medicaid, and private health plans [19]. In addition, the use of multivariable linear regression helps to detangle the contribution of obesity from overall metabolic burden while controlling for age and sex.

## Conclusions

In this analysis of a large cardiovascular outpatient quality improvement registry, obesity was common among patients with HFpEF and was not always co-occurring with metabolic syndrome. In addition, we found that patients with obesity-related HFpEF had lower odds of other cardiac comorbidities, providing evidence that HF in this population can occur in the absence of other clinical burdens. Ongoing clinical trials will provide greater insight into the potential causal relationship between obesity and HFpEF.

### Electronic supplementary material

Below is the link to the electronic supplementary material.

Supplementary Material 1

Supplementary Material 2

## Data Availability

The data that support the findings of this study are available from Veradigm but restrictions apply to the availability of these data, which were used under license for the current study, and so are not publicly available. Data are however available from the authors upon reasonable request and with permission of Veradigm.

## Abbreviations

BMI: Body mass index
CI: Confidence interval
HF: Heart failure
HFpEF: Heart failure preserved ejection fraction
HFrEF: Heart failure reduced ejection fraction
HIPAA: Health Insurance Portability and Accountability Act of 1996
IQR: Interquartile ranges
LVEF: Left ventricular ejection fraction
TIA: Transient ischemic attack
